# ATP, an attractive target for the treatment of refractory chronic cough

**DOI:** 10.1007/s11302-022-09877-z

**Published:** 2022-06-21

**Authors:** Mengru Zhang, Dominic L. Sykes, Laura R. Sadofsky, Alyn H. Morice

**Affiliations:** 1 Respiratory Research Group, Hull York Medical School, Cottingham, UK; 2 Department of Pulmonary and Critical Care Medicine, Tongji Hospital, Tongji University School of Medicine, Shanghai, China

**Keywords:** Refractory chronic cough, Cough hypersensitivity, ATP, P2X3 antagonists, Gefapixant, Antitussive

## Abstract

Chronic cough is the most common complaint in respiratory clinics. Most of them have identifiable causes and some may respond to common disease-modifying therapies. However, there are many patients whose cough lacks effective aetiologically targeted treatments or remains unexplained after thorough assessments, which have been described as refractory chronic cough. Current treatments for refractory chronic cough are limited and often accompanied by intolerable side effects such as sedation. In recent years, various in-depth researches into the pathogenesis of chronic cough have led to an explosion in the development of drugs for the treatment of refractory chronic cough. There has been considerable progress in the underlying mechanisms of chronic cough targeting ATP, and ongoing or completed clinical studies have confirmed the promising antitussive efficacy of P2X3 antagonists for refractory cough. Herein, we review the foundation on which ATP target was developed as potential antitussive medications and provide an update on current clinical progresses.

## Introduction

Cough is an important physiological protective reflex to avoid aspiration into the airways and to maintain airway patency. However, in some individuals excessive, dry or minimally productive cough becomes problematic leading to decrement in quality of life [1]. The commonly adopted definition of chronic cough (CC) in adults is a cough lasting for at least 8 weeks [2]. Following the current cough guidelines, the majority of patients with chronic cough can have identifiable causes and some may respond to common disease-modifying therapies. However, there are many patients whose cough lacks effective aetiologically targeted treatments or remains unexplained after thorough assessments. A variety of terms have been used to describe this condition which is referred to (synonymously) as *refractory chronic cough* (RCC) or *unexplained chronic cough* (UCC) in recent literature [3, 4].

RCC has been reported as a common clinical problem, with a worldwide prevalence of approximately 10% [5]. In specialist cough clinics, the prevalence may be up to 59.1% [6]. These patients often share a common feature in that their troublesome cough is often triggered by levels of stimuli such as perfumes and change in temperature which ordinarily would not cause cough in healthy people. This is characterized as allotussia to innocuous stimuli, abnormal sensations in the throat (laryngeal paresthesia), and increased response to tussive stimuli (hypertussia). Collectively, this is known as *cough hypersensitivity syndrome* (CHS) [7, 8]. This common clinical presentation is thought be due to dysregulation of neuronal pathways arising from the airways. Dysregulation may include both peripheral and central pathways. This latter provides the foundation for the importance of neuromodulators in treating RCC, such as currently used gabapentin and baclofen [9]. At present, it is unclear whether these medications are likely to have a non-specific antitussive effect in RCC by inhibiting the sensitized central cough pathways. Their efficacy is poor being only around 50% even in uncontrolled studies, and with prominent drug-related adverse events such as sedation [10, 11]. A single agent, low-dose morphine, has been shown in a placebo-controlled RCT to be efficacious in about a third of patients with RCC [12].

A significant body of evidence has indicated the peripheral mechanisms are of importance in cough hypersensitivity. Inhalation challenge studies have demonstrated increased cough reflex cough sensitivity from peripheral stimulation. Inhalation of capsaicin, cinnamaldehyde, allyl-isothiocyanate indicates that peripheral sensor receptors such as transient receptor potential vanilloid 1 (TRPV1), transient receptor potential vanilloid 4 (TRPV4), and transient receptor potential ankyrin 1 (TRPA1) receptors have an important role in upregulating the cough sensitivity. However, their antagonists did not show efficacy in RCC clinical trials [13–17]. RCC patients are thus bereft of effective treatments and undergo significant physical, psychological, and socio-economic stress, with consequent serious negative impacts on the quality of life (QoL) [4, 18]. The treatment of RCC is currently a therapeutic black hole and there is an urgent need for safe, effective, non-sedating medications for the treatment of this common condition.

**Fig. 1:**
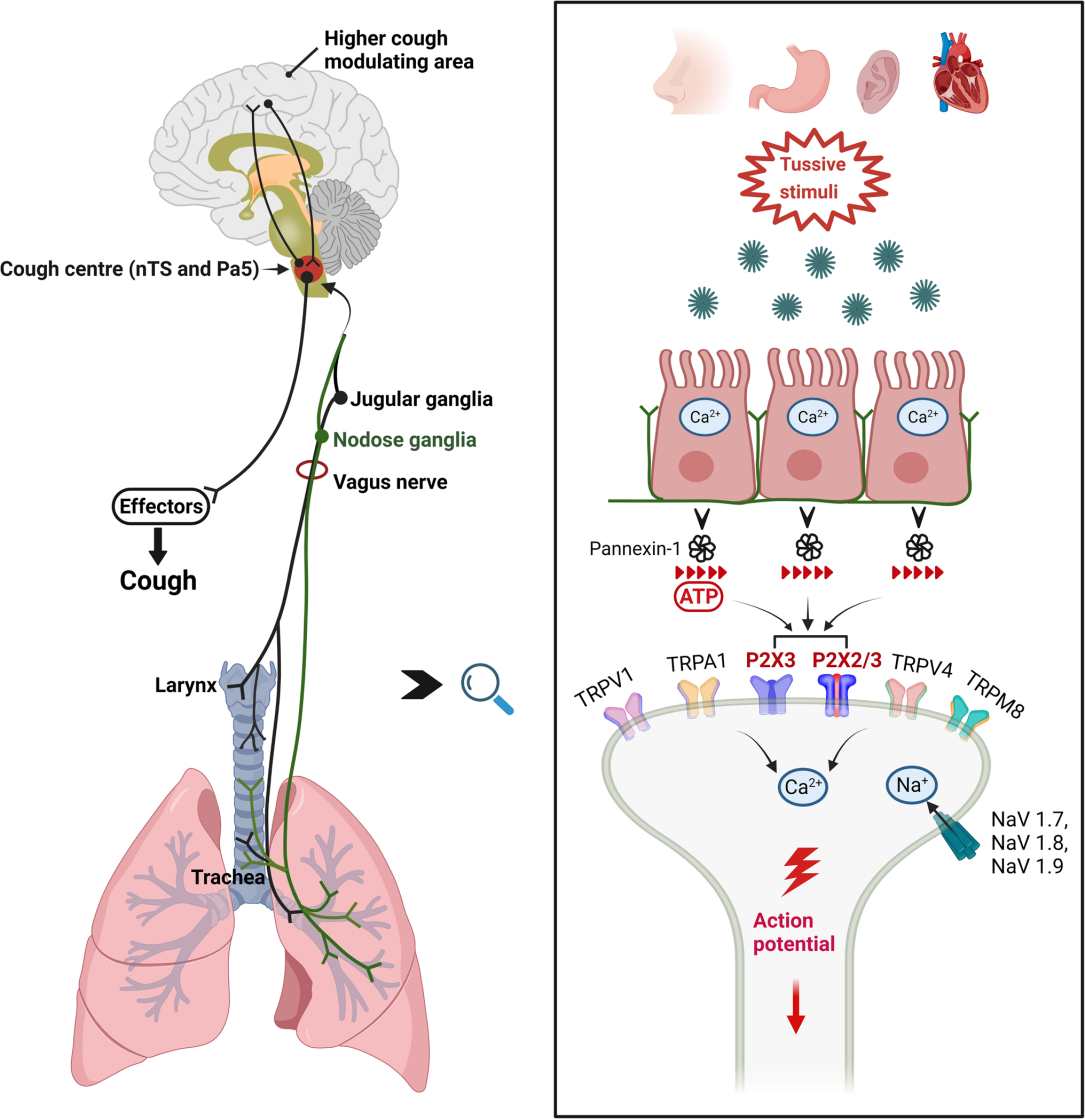
Current understanding of the neural processes in the cough reflex. Tussive stimuli from various sources can increase the calcium influx, leading to ATP release from the open pannexin-1 channel. This in turn activates the P2X3 and P2X2/3 receptors on sensory neurones within the airway mucosa. Other ion channels (TRPV1, TRPA1, TRPV4, TRPM8) on nociceptor terminals originating from jugular or nodose ganglia are activated by irritants or inflammatory reactions. These processes combine to produce an action potential, which is carried along the vagus nerve to cough centre (nTS and Pa5) and onwards to the central nervous system to regulate cough reflex. This is a gross oversimplification of an extremely complex neural pathway. The precise mechanism of cough still remains to be elucidated, particularly the mechanism producing the hypersensitization seen in patients with chronic cough. Other pathways and receptor systems are likely to be revealed by future work. ATP: adenosine triphosphate; nTS: nucleus of the solitary tract; Pa5: paratrigeminal nucleus; Ca^2+^: calcium; Na^+^: sodium; TRPV: transient receptor potential vanilloid; TRPA: transient receptor potential ankyrin; TRPM: transient receptor potential melastatin; NaV: voltage-gated sodium channel

Recently, knowledge of neural pathways in RCC has been advanced through focus on adenosine triphosphate (ATP) activating purinergic P2X3 receptors (Fig. 1). Great progress has been made in the exploration of P2X3 antagonists as potential antitussive medications. Herein, we review the foundation on which ATP target was developed as potential antitussive medications and provide an update on current clinical progresses.

## ATP as a key modulator of the cough reflex

ATP was long known as an intracellular energy source involved in metabolic processes of all cells. When released extracellularly, it is hydrolyzed to AMP and then adenosine by extracellular nucleotidases. In 1972, Geoffery Burnstock proposed that ATP and related nucleotides could be neurotransmitters co-released with noradrenaline, which subsequently acted on non-adrenergic and non-cholinergic nerves [19, 20]. This new concept was not widely accepted initially, but gradually evidence accumulated suggesting a role for the purinergic signalling. The role of ATP was confirmed 20 years later when the first P2X subtype was cloned [21]. In recent years, many studies have focused on the role of extracellular signalling by ATP released in response to cell damage—the so-called alarmin concept. The demonstration of P2X receptors on the peripheral afferent nerves supports the role of ATP as a short-term signalling molecule in disorders in multiple different systems, such as visceral pain, bladder incontinence, hypertension, and chronic cough [22, 23].

That the release of extracellular ATP plays a major role in RCC has been now well established. After release from non-neuronal cells such as the injured airway epithelium and immune cells by cell lysis or through pannexin channels on the plasma membrane, ATP can stimulate afferent sensory nerves and release other proinflammatory cytokines driving further inflammation [24–26]. This can induce acute peripheral sensitization, as revealed by the acute cough response to ATP inhalation challenge in healthy volunteers (HV). Patients with RCC exhibited a greater degree of response to ATP inhalation [27, 28]. ATP also showed a more potent effect than AMP on inducing cough and bronchoconstriction in asthmatic patients [29, 30]. With aerosolized ATP, more dyspnea, cough, and throat irritation can also be observed in smokers and patients with COPD, when compared to healthy subjects [31]. Furthermore, in bronchoalveolar lavage fluid (BALF) of chronic smokers and ex-smokers with chronic obstructive pulmonary disease (COPD), elevated ATP concentrations were detected, indicating ATP could contribute to the symptoms and inflammation in the pathogenesis of some chronic respiratory diseases [32]. Similar findings can be seen in asthmatic humans [33]. Given that cough is a major symptom of airway disease, this suggests the likelihood that ATP could directly or indirectly enhance the cough reflex in diverse respiratory conditions.

Some animal studies help to verify the plausibility of the involvement of ATP in cough. However, the effect of ATP appears to manifest a significant species difference. Generally, most of the peripheral sensory fibres innervating the respiratory tract are originated from the vagus, and relay through two distinct ganglia referred to as the nodose and the jugular ganglia. The peripheral stimuli activate C fibres which respond to a wide range of chemical and mechanical stimuli, signalling to respiratory central neural circuits [1]. A rapid ATP bolus administration into right atrium or pulmonary artery of a canine model could only stimulate the afferent capsaicin-sensitive vagal C fibre terminals and trigger a vagal reflex [34]. In mice, ATP can activate both capsaicin-sensitive and capsaicin-insensitive C fibres [35]. Given the difficulty in mimicking cough in models such as mice or rats [36], most early cough challenge studies were conducted using guinea pigs. In guinea pigs, capsaicin-insensitive nodose ganglia neurons, which terminate intrapulmonarily, can be activated by ATP (some specialized Aδ fibres, which are commonly named cough receptors, terminate in the larger airways and do not have action potential firing in respond to ATP); but the jugular ganglia neurons, which terminate in the larynx, trachea, and mainstem bronchi, are all ATP-insensitive [37–39]. The non-hydrolyzable form of ATP, α, β-methylene ATP, was found to have a direct and receptor-dependent effect on the rapidly adapting receptors (RARs, also called Aβ fibres); however, it failed to evoke cough in conscious guinea pigs [37]. Later, Kamei and his colleagues confirmed that the extracellular ATP, by itself, did not elicit cough, with an acute guinea pig cough model in vivo, but caused increased coughing in response to citric acid. They also found that the ATP-induced cough sensitivity to citric acid was not changed by the desensitization of C fibres, but capsaicin-induced coughs were reduced. Based on the fact that citric acid stimulates both vagal C fibres and RARs, whereas capsaicin appears to only act on C fibres, they proposed the likelihood that RARs were involved in the pathway of ATP action in cough [40].

Cough is usually evoked by stimuli from larynx, trachea, and large airways, which have the jugular chemosensitive terminals and the specialized nodose Aδ fibre terminals (cough receptors). It is puzzling that all these terminals are ATP-insensitive. In recent years, jugular airway nociceptors, rather than nodose ganglia, were thought to play a critical role in the induction and sensitization of cough in non-human species, which may in part be in agreement with the weak tussigenic feature of ATP by itself [41, 42]. However, this does not conflict with the theory that ATP sensitizes the irritated cough sensor and exacerbates cough.

Taken together, these in vivo animal studies give confusing pictures of the cough reflex pathways in different animal species and give little insight into the role of ATP in RCC.

## Strength of rationale for P2X3 purinergic receptor inhibition

Purinergic receptors are divided into 2 classes—A receptors (also termed P1R), whose ligand is adenosine, and P2 receptors (P2R, which primarily recognize nucleotides usually AMP and ATP). The P2 receptors contain two further subtypes: G protein–coupled receptors (P2Y, subunits (functional human receptors) are numbered 1, 2, 4, 6, 11, 12, 13, and 14) and ligand-gated ion channels (P2X, homotrimers or heterotrimers of subunits numbered 1–7) [43, 44]. The expression of purinergic receptors and ectonucleotidases varies in different tissues or cells under physiological and pathophysiological situations.

Once ATP is released from the cells under pathological conditions, it will act on P2 receptors as a local mediator in an autocrine or paracrine manner. The releases of these and other distress signals are collectively known as alarmins. Currently, there is a growing understanding of purinergic signalling in almost every system. Since ATP can enhance cough reflex in pulmonary diseases, it is reasonable to assume that the blockade of ATP receptors on vagal fibres could modulate cough hypersensitivity. In vivo studies pointed to the expression of functional P2X receptors on the nodoses neurons projecting C-fibres to the lungs of guinea pigs and canines [34, 39]. In 2005, Kamei and his colleagues found ATP-induced enhanced cough reactivity to citric acid in guinea pigs could be abolished by TNP-ATP, an antagonist of P2X1-4 [40]. Later, in 2006, they reported that the combination of TNP-ATP and reactive blue 2 (a P2Y antagonist) could completely eliminate the histamine-induced increased cough reactivity to citric acid in guinea pigs [45]. Homotrimeric P2X3 receptors (e.g. with three P2X3 subunits) and heterotrimeric P2X2/3 receptors (e.g., with two P2X3 subunits and one P2X2 subunit), which are expressed in both peripheral and central terminals of the vagus, are the most investigated subunits of P2X receptors [46, 47]. P2X3 receptors were first cloned in 1995 and were demonstrated to be located on small nociceptive sensory neurons in dorsal root ganglia (DRG) with lectin IB4 in 1998. Geoffery Burnstock proposed a P2X3 purinergic hypothesis for the initiation of pain in 1996 [48–50]. Later, the P2X3 knockout mice confirmed this receptor’s importance in the field of sensory processing, nociceptive signalling, and hollow organ biology [51–54]. Given the similar hollow organ biological features in the respiratory tract, and the similarity of cough to the physiology of neuropathic pain, RCC was described as a distinct clinical entity and termed neuropathic cough in the last decade [55–57]. Many studies provided the mechanistic evidence for targeting P2X3 as a promising antitussive therapeutic indication. In an ex vivo study, ATP-mediated nodose C fibre activation was found to be inhibited with the P2X2/3 and P2X3 purinoceptors antagonists [58]. The extracellular patch-clamp electrophysiology and single cell RT-PCR analysis revealed the expression of P2X2/3 heteromeric receptors on C fibres derived from nodose ganglion neurons, whereas the jugular neurons primarily expressed homomeric P2X3 receptors [47]. Puzzlingly, in these studies, only heteromeric P2X2/3 receptors were functional, i.e. have a sustained current when activated. In contrast, P2X2 and P2X3 gene knock out mice confirmed that both receptors play an important role in nociceptions [59]. Moreover, the evidence in the preclinical guinea pig cough model that BLU-5937 (a selective P2X3 antagonist) reduced the histamine-enhanced cough reflex to citric acid, and that aerosolized DT-0111 (a selective and effective P2X2/3 antagonist) inhibited ATP-induced bronchoconstriction and cough, intimated the potential for potent antitussive activity of P2X3 antagonist [60, 61]. Taken together, these evidence suggests that P2X2 and P2X3 receptors play different roles dependent on the species and the pathophysiological stimuli provoking their activation.

## P2X receptor antagonists in clinic trials for RCC

P2X receptor antagonists previously widely used in the preclinical studies, such as pyridoxal phosphate-6-azo (benzene-2,4-disulphonic acid) (PPADS), suramin, the dye reactive blue 2, and 2′,3′-O-(2,4,6-trinitrophenyl) ATP (TNP-ATP), have limited potency, selectivity, stability, and poor pharmacokinetics, and have therefore not progressed to clinic trial programmes [62, 63]. A- 317,491 is the first identified competitive and reversible P2X3 antagonist with good systemic bioavailability; however, its water solubility and oral bioavailability are not good [64]. Alternative chemical antagonists with drug-like characteristics such as good oral bioavailability, slow clearance, little blood–brain barrier permeability, and high safety margin have been developed. In the last 7–8 years, there had been an explosion in the clinical application of several novel P2X3 receptor antagonists in RCC (Table 1).

**Table 1:** Current clinical trials of P2X3 recerptor antagonists for the treatment of RCC

Drugs	Study period	Study type	Recruited population	Doses	Treatment courses	Efficacy of primary outcome	Side effects	Publication information
Gefapixant	Phase II	A POC, double-blind, placebo-controlled, crossover RCT	24 RCC	600 mg twice daily	2 weeks	the placebo-adjusted objective daytime cough frequency reduced by 65%	All patients had taste Disturbances, causing withdrawal in 25% subjects	Abdulqawi R, et al. (2015). *Lancet*[65]
	Phase II	A double-blind, 2-period, crossover RCT	24 CC and 12 HV	Single-dose 100 mg	Stumuli (ATP, citric acid, capsaicin, and distilled water) challenges were performed 1, 3 and 5 h after gefapixant	Significant increases in ATP challenge C2 and C5 were observed in CC and HV with gefapixant, which were also found in distilled water C2 and C5 in CC. However, gefapixant had no effect on capsaicin or citric acid challenge	Dysgeusia was the most frequent side effect (75% HV and 67% CC), but not serious	Morice AH, et al. (2019). *Eur Respir J*[28]
	Phase IIa	Two double-blind, placebo-controlled, crossover, dose-escalation RCTs	Study 1 (n = 29 RCC); Study 2 (n = 30 RCC)	Study 1: 50, 100, 150, 200 mg, twice daily; Study 2: 7.5, 15, 30, 50 mg, twice daily	16 days	Reduction of the awake objective cough frequency was maximal at dose ≥ 30 mg	Taste disturbances were dose-dependent	Smith JA, et al. (2020). *Eur Respir J*[66]
	Phase IIb	A double-blind, placebo-controlled, parallel-group RCT	253 RCC	7.5 mg, 20 mg or 50 mg, twice daily	12 weeks	Geometric mean of awake cough frequency was reduced by 22·0% (p = 0·097) with 7·5 mg, 22·2% (p = 0·093) with 20 mg, and 37·0% (p = 0·0027) with 50 mg, relative to placebo, twice daily	Taste disturbances showed a clear relationship with the dose of gefapixant, and occurred in 81% patients with 50 mg gefapixant	Smith JA, et al. (2020). *Lancet Respir Med*[67]
	Phase III	Two global, parallel, double-blind, placebo-controlled RCTs	COUGH-1 (n = 730 RCC); COUGH-2 (n = 1314 RCC)	15 mg or 45 mg, twice daily	12 weeks for COUGH-1 (extension periods of 40 weeks); 24 weeks for COUGH-2 (extension periods of 28 weeks)	45 mg of gefapixant reduced 24-h cough frequency by 18.5% in COUGH-1 and 14.6% in COUGH-2, relative to placebo, while 15 mg of gefapixant, had no significant efficacy	Incidence of taste disturbance was 59.3% in COUGH-1 and 68.9% in COUGH-2, and most of them were tolerated and reversed after cessation of treatment	Muccino DR, et al. (2020). *ERJ Open Res*[68]; McGarvey LP, et al. (2021). *Lancet*[69]
Eliapixant	Phase I	Double-blind, placebo-controlled, ascending dose RCT	47 HV	10 mg, 50 mg, 200 mg, or 750 mg, twice daily	2 weeks	N/A	High doses (200 mg and 750 mg) produced plasma concentrations that cover the predicted therapeutic threshold over 24 h, with good safety and tolerability	Christian F, et al. (2021). *Clinical Pharmacokinetics*[70]
	Phase IIa	Two periods, double-blinded, placebo-controlled, parallel-group RCT	40 RCC	Period A: 2 weeks of placebo followed by 1 week of 10 mg; Period B: 50, 200 and 750 mg twice daily for 1 week per dose level	Seen the left	24-h objective cough frequency significantly reduced at doses ≥ 50 mg, versus placebo (14.8% at 50 mg, 22.6% at 200 mg and 25% at 750 mg)	Mild taste-related side effects occurred in 10%-21% subjects when doses ≥ 50 mg	Morice A, et al. (2021). *Eur Respir J*[71]
	Phase IIb	An international placebo-controlled, double-blind, parallel group, dose-finding RCT	310 RCC	25, 75 or 150 mg, twice daily	12 weeks	Objective cough frequency was reduced by 27% over placebo at dose of 75 mg	Taste-related side effects occured in 24% patients with the highest dose of 150 mg, which were markedly less under lower doses. Most of them were mild or moderate	Lorcan McGarvey, et al. (2021). *Eur Respir J*[72]
BLU-5937	Phase I	Double-blind, placebo-controlled, ascending dose RCTs	Single ascending doses (n = 60 HV); Multiple ascending doses (n = 30 HV)	single ascending doses (50, 100, 200, 400, 800, 1200 mg); Multiple ascending doses of 100, 200, 400 mg, twice daily	7 days	N/A	BLU-5937 was considered to be safe and well tolerated in HV. No complete taste loss at any dose; only one case of mild, transient and sporadic taste alteration at the anticipated therapeutic doses (500-100 mg)	Garceau D, (2020). *Lung*[73]
	Phase IIa	A POC, two-period, crossover, dose-escalation RCT (RELIEF)	69 RCC	25, 50, 100 and 200 mg, twice daily	16 days	Significant Improvements in the awake cough counts were not seen in the intent-to-treat population, but were observed in a pre-planned sub-group analysis of patients with higher baseline cough frequency: awake cough frequencies at baseline of ≥ 20 coughs/h (-23.8%, -19.1%, and -27.3% at 25, 50, and 200 mg, respectively, over placebo) or ≥ 32 coughs/h (-29.0%, -28.8%, -27.1% and -32.1% at 25, 50, 100 and 200 mg, respectively, over placebo)	No complete taste loss; taste-related side effects were infrequent at any dose (6.5%, 9.8%, 10% and 8.6% at 25, 50, 100 and 200 mg, respectively, versus 4.9% with placebo) and were mostly mild in nature	Smith J, et al. (2021). *American journal of respiratory and critical care medicine*[74]; Businesswire A Berkshire Hathaway Company [Internet]. Available from: https://www.businesswire.com/news/home/20200706005125/en/BELLUS-Health-Announces-Topline-Results-Phase-2 [75]
	Phase IIb	A multi-centre, placebo-controlled, parallel arm, dose-finding RCT (SOOTHE)	Main study (n = 249 RCC with baseline awake cough frequency ≥ 45 coughs per hour); exploratory group (n = 61 RCC with a baseline awake cough frequency of ≥ 10 and < 25 coughs per hour)	Main study: 12.5, 50 and 200 mg, twice daily; exploratory group: 200 mg, twice daily	4 weeks	Significant placebo-adjusted reduction of 34% was observed in 24-h cough frequency at 50 mg and 200 mg BID doses	BLU-5937 was well-tolerated with only a few taste-related adverse events (≤ 6.5%)	Bonuccelli CM, et al. (2021). *American journal of respiratory and critical care medicine*[76]. LAVAL, Quebec–(BUSINESS WIRE)–Dec. 13, 2021– BELLUS Health Inc. Available from: https://ir.bellushealth.com/news-releases/news-release-details/bellus-health-announces-positive-topline-results-its-phase-2b [77]
Sivopixant	Phase IIa	A double-blind, placebo-controlled, crossover, multicentre RCT	31 RCC	150 mg, once daily	2 weeks	Reductions in the average hourly objective coughs in day-time (primary outcome) failed to achieve statistical significance (p = 0.0546) but were significant in 24 h (secondary outcome, p = 0.0386)	Only 2 cases of mild taste disturbance (6.5%) were observed	Niimi A, et al. (2021). *Eur Respir J*[78]
	Phase IIb	A dose-finding, double-blind, placebo-controlled, parallel-group, multicentre RCT	372 RCC	50, 150 or 300 mg, once daily	4 weeks	The statistically significant placebo-adjusted change in 24-h cough frequency (primary efficacy endpoint) was not met at any dose	Incidence of taste-related side effects were dose-dependently (2.0%, 13.6%, 33.0% at 50, 150 or 300 mg, respectively, versus 2.9% with placebo)	Ishihara H, et al. (2020). *European Respiratory Journal*[79]; SHIONOGI & Co., Ltd. [cited 2021 Dec 30]. Available from: https://www.shionogi.com/content/dam/shionogi/global/investors/ir-library/presentation/2021/e_210929_3(3).pdf [80]
Filapixant	Phase IIa	A double‐blind, placebo‐controlled, two‐way crossover RCT	23 RCC	20, 80, 150, or 250 mg, twice daily	16 days	Significant reductions in objective 24-h coughs per hour and cough severity were observed at doses ≥ 80 mg	Taste‐related side effects were mild‐to‐moderate, and occurred in 4%, 13%, 43%, and 57% of patients at 20, 80, 150, and 250 mg dose, respectively, vesus 12% with placebo	Friedrich C, et al. (2020). *European respiratory journal*[81]

*CC*, chronic cough; *C2*, lowest concentration of inhaled solution required to evoke ≥ 2 coughs; *C5*, lowest concentration of inhaled solution required to evoke ≥ 5 coughs; *HV*, healthy volunteers; *POC*, proof-of-concept; *RCT*, randomized controlled trial; *RCC*, refractory chronic cough.

### Gefapixant

Gefapixant (previously known as AF-219 and MK-7264) was named after Geoffery Burnstock and is the first in class P2X3 and P2X2/3 receptor-selective antagonist. This molecule, as with all other antagonist in development, is reversible and is an allosteric (non-competitive) antagonist. It was firstly developed by Roche and subsequently licenced to Afferent who undertook the initial successful clinical studies. Merck then purchased the rights for over one billion dollars. It has a good pharmacokinetic profile and low potential to cause a clinically relevant drug-drug interaction [82–85]. A range of clinical studies have been undertaken leading to two phase III trials which have recently been successfully completed. They confirmed its antitussive efficacy and regulatory approval is expected shortly for the treatment of RCC in practice [86].

The phase I study examined the pharmacokinetics, safety, and tolerability of gefapixant by profiling a very large range dose and exposure levels in HV and subsequently confirmed its high oral bioavailability and resistance to metabolic degradation [62]. The initial proof-of-concept (POC) phase II study of AF-219 suggested its promising efficacy in RCC [65]. In this randomized, double-blind, placebo-controlled, crossover study in 24 RCC patients, gefapixant produced significant reductions in objective cough frequency. However, the high dose administered (600 mg twice daily) produced marked taste disturbances (hypogeusia or dysgeusia), which caused withdrawal in 25% subjects. In another double-blind, randomized, 2-period, crossover phase II study [28], which was conducted on 24 CC patients and 12 HV, a lower single-dose gefapixant 100 mg inhibited ATP-evoked cough in CC and HV, as well as distilled water–evoked cough in CC, but had no effect on capsaicin or citric acid challenge. Median cough frequency was reduced by 42% with gefapixant over placebo in CC subjects. This suggests the underlying role of TRPV4/ATP-mediated P2X3 receptor activation in the pathophysiology of chronic cough as a peripheral target. The preservation of the protective irritant-induced cough demonstrated in this study is an important safety signal and subsequent larger studies have failed to demonstrate any excess of aspiration pneumonia with P2X3 antagonist. Taste-related issues were again the most common adverse events, during which dysgeusia was reported in 75% HV and 67% CC.

The notable taste adverse events are thought to be due to the relatively poor selectivity of gefapixant for the P2X3 receptor over the P2X2/3 heterotrimer. This may lead to unmasking effects in clinical trials and inhibit medication compliance. Therefore, the exploration of the optimal cough relief of P2X3 antagonist with a diminished or even eliminated side effect on taste was the objective in the subsequence phase II studies. Two crossover-designed randomized dose-escalation studies (study 1: 50–200 mg, twice daily; study 2: 7.5–50 mg, twice daily) were reported in the *European Respiratory Journal* in 2020, and allowed the calculation of optimal dosing [66]. Reduction of the awake cough frequency was maximal at dose ≥ 30 mg, two times daily, which was far lower than the dose in the POC. Taste disturbances were also dose-dependent. On the basis of these findings, a randomized, double-blinded, controlled, parallel-group, phase IIb study was performed, evaluating the efficacy of gefapixant at one of three doses of 7.5 mg, 20 mg, or 50 mg, twice daily, over 12 weeks [67]. With the dose of 50 mg, geometric mean of awake cough frequency was reduced by 37%, relative to placebo, and with marked improvements in cough-related assessments; taste disturbances occurred in 81% patients although at a much-reduced severity.

Two global, parallel, double-blind, randomized placebo-controlled phase III trials (COUGH-1 and COUGH-2) were designed [68] and completed in March 2020. Subjects were administrated either placebo, gefapixant 15 mg or 45 mg, twice daily, in a ratio of 1:1:1. Study period differed, 12 weeks for COUGH-1 (extension periods of 40 weeks) and 24 weeks for COUGH-2 (extension periods of 28 weeks). A total of 2044 RCC patients were recruited, in COUGH-1 (*n* = 730) and COUGH-2 (*n* = 1314). Gefapixant at the dose of 45 mg reduced 24-h cough frequency by 18.5% in COUGH-1 and 14.6% in COUGH-2, relative to placebo. The placebo response was considerably higher in these phase III studies with a reduction of 24-h cough count greater than 50% compared to the approximate 30% seen in phase II studies. Taste disturbance remained to be the most common adverse events, with the incidence of 59.3% in COUGH-1 and 68.9% in COUGH-2, with most being tolerated and reversing after cessation of treatment. However, 15 mg of gefapixant, two times daily, had no significant efficacy compared to placebo, which presumably swamped any treatment effect. Thus, 45 mg of gefapixant was finally proven to be an effective antitussive option for these RCC patients who had a mean duration of cough greater than 10 years. The reports of COUGH-1 and COUGH-2 were published in *The Lancet* [87].

In a trial of idiopathic pulmonary fibrosis cough [88], 50 mg of gefapixant, two times daily, demonstrated a poor efficacy in awake cough frequency and a similar incidence of efficacy-unrelated taste disturbance (78.7%) [89]. This may suggest the heterogeneity in the underlying etiology in disease-specific cough and RCC although there were a number of methodological problems in this study.

Because of the allosteric nature of P2X3 antagonists, their roles in mediating taste signalling or cough hypersensitivity has not been well established as yet [90]. P2X2/3 heterotrimeric receptors were speculated to play a dominant role in taste signalling with evidence that the chorda tympani and glossopharyngeal nerves failed to response to all taste qualities in P2X2 and P2X3 double knockout mice, but did not exhibit such severe taste disturbance in P2X2 or P2X3 single knockout mice [91, 92]. Given that gefapixant demonstrated approximately threefold low degree of selectivity for P2X3 homotrimers over P2X2/3 heterotrimers [57, 84], a new generation of antagonists with higher selectivity to P2X3 are under clinical development.

### Eliapixant (BAY-1817080)

A novel, highly selective (confirmed by patch clamp studies) P2X3 receptor antagonist, eliapixant, was developed by Bayer (BAY-1817080)[82, 93]. In vivo studies showed its potential efficacy in nerve hypersensitization [93]. Eliapixant has been reported for its good tolerability in HV after single and multiple dosing [70]. Promising efficacy for RCC was shown in a phase IIa study [71]. In this randomized, placebo-controlled, double-blinded, crossover study, eliapixant was administered twice daily in two treatment periods: 2 weeks of placebo followed by 1 week of 10 mg and escalating doses of 50, 200, and 750 mg, each for 1 week. Forty RCC patients were assessed with the change in objective 24-h cough frequency as the primary endpoint. Doses ≥ 50 mg demonstrated a 15% reduction in cough frequency compared with placebo, with a lower incidence of taste-related side effects of 10–21% (all mild). Recently, Bayer reported the most current encouraging results of the international placebo-controlled, randomized, double-blind, parallel group, phase IIb dose-finding study of eliapixant in RCC at the European Respiratory Society (ERS) International Congress 2021 [72]. A total of 310 participants were administrated orally with either 25, 75, or 150 mg of eliapixant or placebo tablets, twice daily, for 12 weeks. Seventy-five-milligram dose of eliapixant twice daily could reduce objective cough frequency by 27% over placebo, with the majority of side effects considered mild or moderate. Taste-related side effects were reported in 24% of patients with the highest test dose of 150 mg, which were markedly less under lower doses. Taken together, these studies confirmed a lower incidence of taste-related issues with eliapixant at effective therapeutic doses. That eliapixant has demonstrated efficacy in RCC confirms the hypothesis that the P2X3 antagonists, as a class, have an important role in the treatment of this previously intractable condition. Despite the promising clinical trial results with eliapixant clinical trial, development has been suspended by Bayer because of a risk of hepatotoxicity (elevated transaminases) seen in a small number of patients exposed to the 150-mg dosage.

#### BLU-5937

BLU-5937 is another potent non-competitive antagonist stereoselective to P2X3 homotrimeric receptor developed by Bellus Health. It exhibited excellent drug-like characteristics in preclinical studies and showed potential efficacy with limited or no taste disturbance in animal cough model [60]. The randomized, double-blind, placebo-controlled phase I study recruited 90 HV to assess the safety, tolerability, and pharmacokinetic profile of BLU-5937 [73]. During the administration of single ascending doses (50, 100, 200, 400, 800, 1200 mg) or doses of 100, 200, 400 mg, twice daily, for 7 days, BLU-5937 presented excellent pharmacokinetic and safety/tolerability profiles, with only one case of mild, transient, and sporadic taste alteration at the anticipated therapeutic doses (500–100 mg). Recently, the top-line results from the POC phase IIa randomized, double-blinded, placebo-controlled, two-period, crossover, dose-escalation study (the RELIEF trial) of BLU-5937 in RCC patients have been reported at the American Thoracic Society International Conference 2021 [74]. Sixty-nine RCC participants were randomized to 16-day treatment (25, 50, 100, and 200 mg, twice daily) or matching placebo, with dose escalation every 4 days, then were crossed over after a 10–14-day washout. This trial was terminated early due to COVID-19 limitations and failed to reach significant reductions in the awake cough counts in the intent-to-treat population. However, significant reductions were observed in a pre-planned sub-group analysis of patients with higher baseline cough frequency: awake cough frequencies at baseline of ≥ 20 coughs/h (− 23.8%, − 19.1%, and − 27.3% at 25, 50, and 200 mg, twice daily, respectively, over placebo) or ≥ 32 coughs/h (− 29.0%, − 28.8%, − 27.1%, and − 32.1% at 25, 50, 100, and 200 mg, twice daily, respectively, over placebo). According to data presented by Bellus Health [75], taste-related side effects were infrequent at all dose levels, which were 6.5%, 9.8%, 10%, and 8.6% at 25, 50, 100, and 200 mg, respectively, versus 4.9% with placebo, and were mostly mild in nature. No patients reported complete taste loss. Higher cough counts were assumed to be the best available clinical indicator of cough hypersensitization via the P2X3 pathway; however, conflicting results have been seen in other studies and this may represent a statistical artefact akin to regression to the mean. These data moved forward BLU-5937 to an adaptive phase IIb trial for RCC patients with higher cough counts [76, 94]. This is a multi-centre, randomized, double-blind, parallel arm dose-finding study (the SOOTHE trial), and included a placebo run-in period and stratification by baseline cough frequency. A total of 240 patients with a baseline awake cough frequency ≥ 25 coughs/h were randomized to the three active treatment arms of BLU-5937 (12.5, 50, and 200 mg, twice daily, 1:1:1:1) or placebo for 4 weeks after a single-blind run-in period. An exploratory population of participants (*n* = 60) with baseline awake cough frequencies between 10 and 25 coughs/h will be randomized to placebo and BLU-5937 200 mg twice daily treatment arms (1:1). The primary endpoint is the reduction in objective 24-h cough frequency versus placebo. In December 2021, Bellus Health announced the positive top-line results of this study [77]. Significant placebo-adjusted improvement of 34% was observed in 24-h cough frequency (the primary efficacy endpoint) at 50 mg and 200 mg BID doses with a few taste-related adverse events (≤ 6.5%).

### Sivopixant (S-600918)

Sivopixant (also called S-600918), firstly reported by Shionogi, is a newly developed antagonist with favourable pharmacokinetic profiles and higher selectivity to P2X3 over P2X2/3 trimeric homomer [95]. Its promising antitussive efficacy with limited taste-related side effects for RCC has been demonstrated in a POC phase IIa, randomized, double-blind, placebo-controlled, crossover, multicentre study [78]. In this study, 31 RCC patients were randomized to oral sivopixant 150 mg or placebo once daily for 2 weeks, then crossed over for another 2 weeks after a 2–3-week washout. The placebo-adjusted cough reductions in the average hourly objective coughs in day-time (primary outcome) and in 24 h (secondary outcome) were − 31.6% (*p* = 0.0546) and − 30.9% (*p* = 0.0386), respectively, accompanied with the significant improvement in health-related quality of life measured with Leicester Cough Questionnaire but not in Visual Analogue Scoring. Only 2 cases of mild taste disturbance (6.5%) were observed. The authors attributed the lack of statistical significance in primary outcome to the insufficient sample size. However, in the subsequent phase IIb dose selection study (ClinicalTrials.gov identifier: NCT04110054), which was conducted in 372 RCC subjects assigned to 50, 150, or 300 mg of oral S-600918 or matched placebo for 4 weeks, the statistically significant placebo-adjusted change in 24-h cough frequency (primary efficacy endpoint) was not met at any dose. Incidence of taste-related side effects was dose dependent (2.0%, 13.6%, 33.0% at 50, 150, or 300 mg, respectively, versus 2.9% with placebo) [79, 80]. These top-line primary data were reported at Shionogi R&D Day, September 29th, 2021. Next steps are under consideration.

In this study, as with the difference between gefapixant phase II and the phase III studies, a larger placebo effect was seen. In both cases, earlier recruitment had been limited to specialized centres experienced in dealing with RCC patients who have usually undergone multiple failed treatment trials. Placebo response may be greater in treatment-naïve patients and centres. This is an important consideration for the design of future multicentre phase 3 studies where correct patient selection will be vital in demonstrating efficacy over placebo response.

### Filapixant (BAY-1902607)

Filapixant, also named BAY-1902607, is another new P2X3 antagonist with high selectivity, developed by Bayer. Currently, it has been investigated in a phase I/II trial in RCC in Netherlands and UK (p.o.) (NCT03535168) (EudraCT2018-000,129–29) [96]. No recent reports of development were identified for phase I development in cough (in volunteers) in Germany (p.o., tablet). Phase I study has been reported as completed on ClinicalTrials.gov, but data have not yet been posted or published. Primary data of efficacy and side effects from phase II POC study have been presented at the ERS International Congress 2020 [81]. Twenty-three RCC patients were randomized to ascending doses of BAY1902607 (20, 80, 150, or 250 mg, twice daily, 4 days each) or placebo in 2-way crossover. The significant decreases in objective 24-h coughs per hour and cough severity were observed at doses ≥ 80 mg. Taste-related side effects were mild-to-moderate and dose-dependent (4–57% with BAY-1902607 versus 12% with placebo). Despite the high selectivity to P2X3, BAY-1902607 had fairly high impact on taste. The complete data is awaited.

### Other antagonists for P2X3 receptors

DT-0111 (Aspirex™), being developed by Danmir Therapeutic, LLC., is a novel, small, water-soluble molecule that acts as a selective antagonist at P2X2/3 receptors. In vitro and in vivo POC studies have demonstrated its potential to be an inhalation drug candidate for ATP-related pulmonary diseases such as chronic obstructive pulmonary disease (COPD) and chronic cough [61]. In addition, Obrecht et al. identified aurintricarboxylic acid (ATA) as a nanomolar-potency allosteric antagonist of P2X3 and P2X1 receptors with weak inhibition to P2X2/3 receptors [97]. However, the clinical evidence for their antitussive effects has not yet been studied.

## Other purinergic receptor targets with promising antitussive effect

P2X4 receptor seems to be an interesting target in modulating cough reflex. P2X4 and P2X4/P2X6 heterotrimers are moderately expressed in the lung [98]. In guinea pigs, ATP-induced enhancement of the number of citric acid–induced coughs was ameliorated by exposure to TNP-ATP (an antagonist of P2X1–4 receptors), but without response to PPADS (an antagonist of P2X1,2,3,5,7 receptors, but not of P2X4); thus, P2X4 receptor was thought to be involved in the ATP-induced enhancement of the cough reflex sensitivity [40]. In vitro and in vivo studies provided evidence that P2X4 is the predominant subunit of P2X expressed in secretory airway epithelial cells and its overexpression could be seen in conditions of chronic inflammation, mucous metaplasia, and hyperplasia [99]. Moreover, PSB-15417, a potent, brain-permeable allosteric P2X4 receptor antagonist for human, rat, and mouse, showed high efficacy in rat models of neuropathic pain, which shares the similar underlying mechanisms with RCC [44, 100]. Another P2X4 receptor antagonist, NC-2600, is currently in clinical trials for chronic neuropathic pain in Japan [101]. P2X7 receptor is also closely associated with neuropathic pain, which has been proved in the spinal cord levels and amygdala [102, 103]. However, the correlation between P2X7 and RCC is lack of sufficient direct evidence, except for the role of P2X7 in mediating ATP efflux [104].

In terms of P2Y receptors, which are expressed in almost all epithelial cells and responsible for fluid control and electrolyte transport, the submits of P2Y2, P2Y4, P2Y6, and P2Y14 are relatively strongly expressed in epithelial and glandular cells of lungs to regulate the physiological functions of respiratory system [98, 105, 106]. The upregulation of P2Y4 and P2Y6 have been detected in allergic bronchospasm, which may lead to an increased production of endogenous ATP via exocytosis following P2Y4 activation, or intensify the inflammatory response via raising inflammatory factors following P2Y6 activation [107–109]. Moreover, combined with TNP-ATP, exposure to reactive blue 2, a P2Y receptor antagonist, could completely reduce histamine-induced increased coughs to citric acid; thus, P2Y receptors were assumed to play partial role in ATP-induced cough hyperreactivity [45]. However, further study to confirm this assumption is warranted.

In the long history of antitussive drug development, there has been many promising targets which have demonstrated efficacy in animal models. A good example is that the TRP receptors and the TRPV1 receptor agonist capsaicin have been long used in cough challenge models. Highly specific TRPV1 antagonist was developed which showed excellent efficacy in capsaicin challenge models demonstrating target engagement. However, these agents have showed no efficacy in RCC [13, 14] and indeed a TRPV4 antagonist was actually showed to provoke cough in RCC [17]. Caution is therefore required in interpreting the results of the preclinical studies of other purinergic receptor targets.

Taken together, given the breakthroughs in P2X3 receptors and taste-related limitations in patients, new drugs with antagonistic activity for other purinergic receptor targets with possible antitussive effect should be taken into consideration.

## Conclusions and perspectives

RCC is a complicated neurobiological process with marked heterogeneity, and which involves multiple peripheral and central neural pathways [110]. Efficacy of the available medications varies from patient to patient, and it seems unlikely to expect a complete elimination of cough symptoms with a single agent. Currently, none of the clinical trials, whether P2X3 antagonists or other neuromodulators, has achieved this goal. Future clinical management of chronic cough will almost certainly require polymodal therapeutic approaches. The antitussive effects of P2X3 antagonists, which suppress the ATP-mediated sensitization of nociceptors, provide potent evidence for ATP-P2X3 pathway in regulating the pathological cough reflex. However, a third of patients fail to respond. Thus, ATP seems unlikely to act as a common mediator for all tussive stimuli which sensitize nerves. Indeed, it is still unclear as to how ATP drives the chronic cough hypersensitization and which sensory neuron pathway is responsible for transmitting the noxious sensation [27, 111]. The combination of further understanding of the neural mechanisms and analysis of clinical variables in RCC patients is essential to identify cough phenotypes in the future. More work is required to identify further therapeutic targets to alleviate this common and chronic disease.

## Data Availability

Not applicable.
